# Long-Term Exposure to Fine Particulate Matter and Academic Performance Among Children in North Carolina

**DOI:** 10.1001/jamanetworkopen.2023.40928

**Published:** 2023-10-31

**Authors:** Pak Hung Lam, Emma Zang, Dieyi Chen, Riyang Liu, Kai Chen

**Affiliations:** 1 The Samuel DuBois Cook Center on Social Equity, Duke University, Durham, North Carolina; 2 Department of Sociology, Yale University, New Haven, Connecticut; 3 Department of Public Health Sciences, Penn State College of Medicine, Hershey, Pennsylvania; 4 State Key Laboratory of Pollution Control and Resource Reuse, School of the Environment, Nanjing University, Nanjing, Jiangsu, China; 5 Department of Environmental Health Sciences, Yale School of Public Health, New Haven, Connecticut

## Abstract

This cross-sectional study analyzes the association of exposure to fine particulate matter and academic performance among school age children in North Carolina.

## Introduction

Elevated exposure to ambient fine particulate matter (PM_2.5_) has been consistently associated with adverse outcomes on children’s test scores.^1^ However, previous research has often relied on relatively small or less representative samples and faced challenges in accounting for unobserved confounders at the individual level.^2^ In this cross-sectional study, we aimed to address these limitations by employing a 2-way fixed-effects model with a large administrative data set in North Carolina.

## Methods

This cross-sectional study followed the STROBE reporting guideline and was approved by the Yale institutional review board with waived informed consent because it was a secondary analysis in accordance with 45 CFR § 46. We obtained student-level administrative data from the North Carolina Education Research Data Center, which included all students in grades 3 to 8 who attended public schools in North Carolina from 2001 to 2018. To ensure comparability across grades and years, following a previous study,^3^ we standardized the test scores in mathematics and reading at the student level as the dependent variable, normalizing the scale to have a mean of 0 and a variance of 1 for each grade-year. More details can be found in Supplement 1. All analyses were performed in R statistical software version 2022.03.0.386 (R Project for Statistical Computing) with packages fixest, splines, and dlnm. A 2-sided *P* < .05 indicated statistical significance and data analysis was conducted from March to September 2023.

Using the school coordinates, we assigned each student the PM_2.5_ concentrations (0.01° × 0.01° resolution^4^) in the previous 12 months prior to the test as the independent variable and the mean daily maximum temperature during summer (June-September) and winter (December-February) (4 km × 4 km resolution^5^) as climate controls. We employed a 2-way fixed-effects regression model by incorporating student- and year-fixed effects to control for all potential time-invariant confounders and some time-varying confounders. To explore potential vulnerable subgroups, we included interaction terms between PM_2.5_ exposure and student characteristics including sex, race and ethnicity (see Supplement 1 for details regarding race and ethnicity categories), and family income. To explore the shape of the concentration-response association, we applied a natural cubic spline for PM_2.5_ with 4 degrees of freedom. To assess the likelihood of model misspecification influencing our main results, we conducted a treatment spatial randomization test, which randomly assigned false PM_2.5_ values to our sample through 2000 iterations.

## Results

Between 2001 and 2018, our data set included 2 801 022 students (1 372 688 female students [49%]; 1 290 680 with low family income [46%]; 77 830 Asian students [2.8%]; 757 289 Black students [27.0%]; 327 133 Hispanic students [11.7%]; 37 194 American Indian students [1.3%]; 92 887 multiracial students [3.3%]) (Table). Each 1 μg/m^3^ increase in PM_2.5_ concentration was found to be associated with a 0.009755 SD (95% CI, −0.010514 to −0.008994 SD) lower standardized end-of-grade mathematics test scores and a 0.006806 SD (95% CI, −0.007592 to −0.006019 SD) lower standardized end-of-grade reading test scores. Our results were also robust to the spatial randomization test (Figure) and the control of grade-fixed effects (Table).

**Table.** Associations of PM_2.5_ Exposure With Academic Performance of Third to Eighth Grade Students in North Carolina and Heterogeneity Within Subgroups

Analysis	Variable outcome, mean (SD) N = 10 346 233^a^	Change in standardized math score (SE)^b^ [95% CI]	*P* Value	Change in standardized reading score (SE)^b^ [95% CI]	*P *Value
**Main analysis** ^c^					
PM_2.5 _concentration, μg/m^3^	10.31 (2.28)	−0.009755 (0.001818) [−0.010514 to −0.008994]	<.001	−0.006806 (0.001144) [−0.007592 to −0.006019]	<.001
Summer maximum temperature	30.2 (1.40)	0.001541 (0.002131) [0.000472 to 0.002609]	.06	−0.001115 (0.001357) [−0.002217 to −0.00001]	.40
Winter maximum temperature	13.10 (2.93)	0.001090 (0.001661) [0.000176 to 0.002003]	.30	0.000119 (0.001105) [−0.000824 to 0.001061]	.11
**Heterogeneity**					
Sex, No. %^a^					
Female	5 086 312 (49)	−0.008712 (0.000535) [−0.009374 to −0.008049]	<.001	−0.000229 (0.000378) [−0.000887 to 0.004277]	.40
Male	5 259 911 (51)	0 [Reference]	NA	0 [Reference]	NA
Family income, No. %^a^					
Low family income	4 770 551 (46)	0.000028 (0.000096) [−0.000089 to 0.000146]	.77	−0.000868 (0.000083) [−0.000992 to −0.000744]	<.001
Non-low family income	5 575 672 (54)	0 [Reference]	NA	0 [Reference]	NA
Race or ethnicity, No. (%)^a^					
Racial or ethnic minority^d^	4 744 363 (46)	−0.014025 (0.000940) [−0.014571 to −0.013479]	<.001	−0.014559 (0.000753) [−0.015109 to −0.014009]	<.001
White	5 601 363 (54)	0 [Reference]	NA	0 [Reference]	NA
**Robustness**^e^					
PM_2.5_ concentration, μg/m^3^	10.31 (2.28)	−0.010337 (0.001809) [−0.011098 to −0.009576]	<.001	−0.007470 (0.001139) [−0.008257 to −0.006683]	<.001

Abbreviation: NA, not applicable.

^a^ Totals reflect the number of test score observations.

^b^ Standard errors cluster at school level.

^c^ The main analysis model is a 2-way fixed effects regression model regressing the standardized test scores on PM_2.5_. Covariates included the mean daily maximum temperature during summer (June-September), and the mean daily maximum temperature during winter (December-February). The estimated coefficient represents the change in standardized end-of-grade mathematics and reading test scores, expressed in standard deviations for every one unit increase in PM_2.5 _concentration (1 μg/m^3^).

^d^ Racial or ethnic minority included Asian, Black, Hispanic, and other (defined as American Indian, multiracial, Native Hawaiian or Other Pacific Islander, unknown, or any other race or ethnicity not otherwise specified).

^e^ The robustness check included grade-fixed effects to the 2-way fixed effect model.

**Figure.**
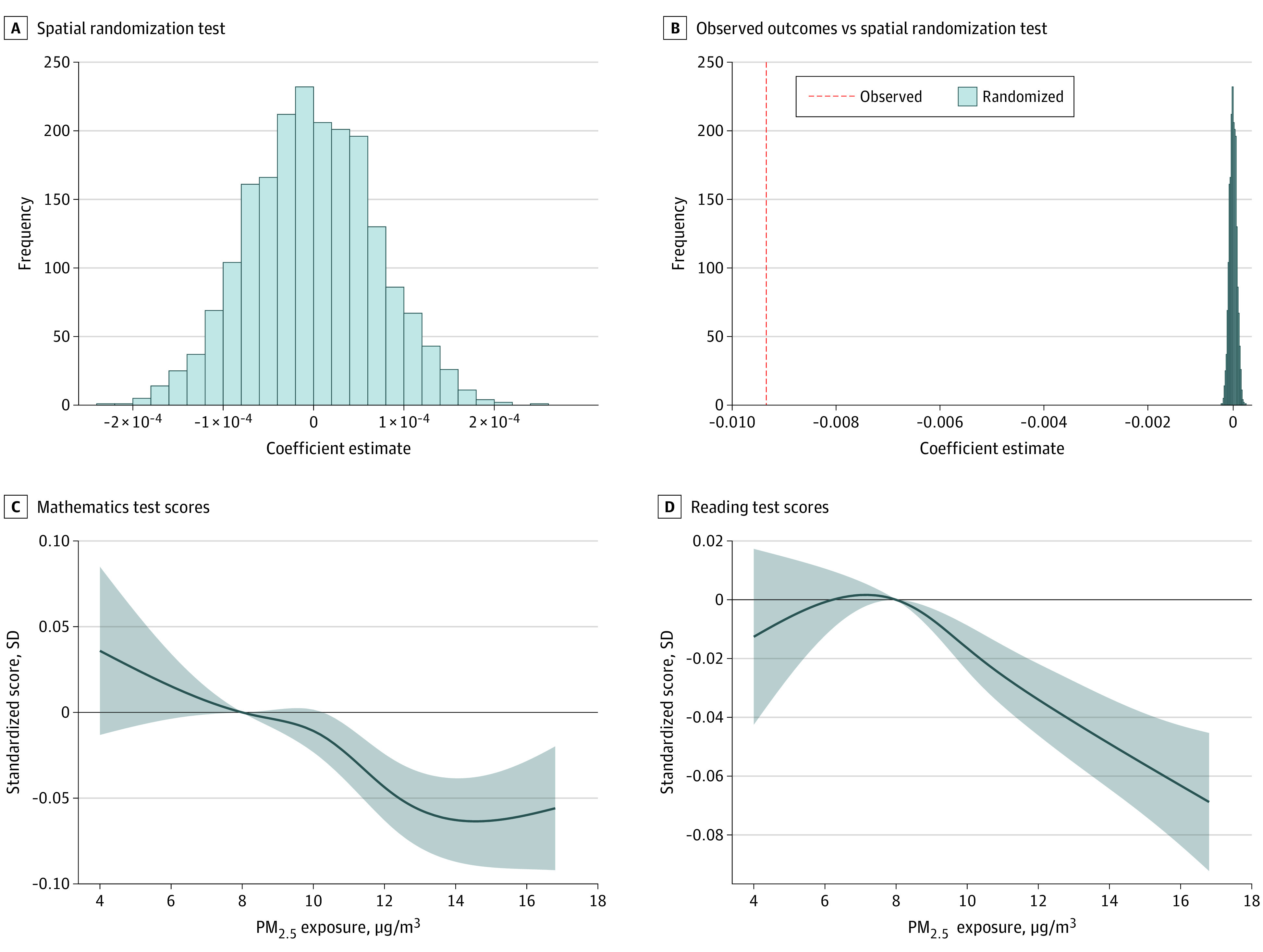
Spatial Randomization Test and Concentration-Response Curves of PM_2.5_ and Standardized Mathematics and Reading Test Scores Panel A displays the results of the spatial randomization test conducted in 2000 iterations, which assessed the likelihood of obtaining the observed outcomes by chance through random assignment of false PM_2.5_ values in the sample. Panel B compares the observed outcomes with the results of the spatial randomization test, allowing for an evaluation of the robustness and statistical significance of the observed outcomes. Panels C and D display the concentration-response association of PM_2.5_ exposure with students’ mathematics test scores (C) and reading test scores (D); they employ a nonlinear model using a natural cubic spline with 4 degrees of freedom to capture potential nonlinear patterns in the associations.

Significant heterogeneous outcomes by sex and race and ethnicity were observed (Table). Girls’ math scores exhibited a considerably higher susceptibility to PM_2.5_ exposure, as were reading scores of students with low family income. Compared with White students, students of minority races and ethnicities tended to be more affected by the same level of PM_2.5_ exposure.

Our nonlinear model revealed a negative association of PM_2.5_ exposure with math scores at concentrations between 10 and 14 μg/m^3^ (Figure), possibly because the majority of our sample fell within this range. PM_2.5_ exposure was associated with a consistent increase in the risk of poor reading performance when exceeding 8 μg/m^3^ (Figure).

## Discussion

Our main findings in this cross-sectional study align with a previous study^6^ that also applied a 2-way fixed effects model, albeit using test score data at the geographic school district level rather than individual level. Our findings are broadly consistent with other previous research.^1^

There are some limitations including the assumption of parallel trend between treatment and control group, no remaining unobserved confounders, potential measurement errors, not considering multiple comparisons, and focus on 1 state. Nevertheless, our study offers an exploratory result to show a significant negative association of long-term PM_2.5_ exposure with children’s academic performance, especially among female students, students with low family income, and students with minority race or ethnicity.

## Notes

Supplement 1.
eMethods.

Supplement 2.
Data Sharing Statement

## References

[1] Stenson C, Wheeler AJ, Carver A, . The impact of traffic-related air pollution on child and adolescent academic performance: a systematic review. Environ Int. 2021;155:106696. doi:10.1016/j.envint.2021.106696 34144475

[2] Stingone JA, McVeigh KH, Claudio L. Association between prenatal exposure to ambient diesel particulate matter and perchloroethylene with children’s 3rd grade standardized test scores. Environ Res. 2016;148:144-153. doi:10.1016/j.envres.2016.03.035 27058443 PMC4874864

[3] Zang E, Tan PL, Cook PJ. Sibling spillovers: having an academically successful older sibling may be more important for children in disadvantaged families. Am J Sociol. 2023;128(5):1529-1571. doi:10.1086/724723 PMC10828899 38298548

[4] van Donkelaar A, Martin RV, Li C, Burnett RT. Regional estimates of chemical composition of fine particulate matter using a combined geoscience-statistical method with information from satellites, models, and monitors. Environ Sci Technol. 2019;53(5):2595-2611. doi:10.1021/acs.est.8b06392 30698001

[5] PRISM Climate Group OSU. PRISM climate data. 2022. Accessed October 11, 2023. https://prism.oregonstate.edu

[6] Lu W, Hackman DA, Schwartz J. Ambient air pollution associated with lower academic achievement among US children: a nationwide panel study of school districts. Environ Epidemiol. 2021;5(6):e174. doi:10.1097/EE9.0000000000000174 34909554 PMC8663889

