# Supplementary material for: Long-Term Exposure to Fine Particulate Matter and Academic Performance Among Children in North Carolina
Source: JAMA Netw Open. 2023 Oct 31;6(10):e2340928. doi: 10.1001/jamanetworkopen.2023.40928 (PMC10618843; doi:10.1001/jamanetworkopen.2023.40928)
Supplement: Supplement 1. — eMethods. [file jamanetwopen-e2340928-s001.pdf]

Supplemental Online Content

Lam PH, Zang E, Chen D, Liu R, Chen K. Long-term exposujre to fine particulate matter and academic performance among children in North Carolina. *JAMA Netw Open*. 2023;6(11):e2340928. doi:10.1001/jamanetworkopen.2023.40928

eMethods

This supplemental material has been provided by the authors to give readers additional information about their work.

© 2023 Lam PH et al. *JAMA Network Open*

eMethods

Our analysis leveraged the North Carolina public school dataset, which is meticulously maintained by the North Carolina Education Research Data Center (NCERDC) at Duke University.

The NCERDC dataset is highly valuable due to its extensive panel data, providing a wealth of information on North Carolina public school students. This dataset allows for the comprehensive tracking of individual student's progress over time at student-level and school-level. All data, including demographic information such as gender, race/ethnicity (Asian, Black, Hispanic, White, and Other (defined as American Indian, multiracial, Native Hawaiian or Other Pacific Islander, unknown, or any other race or ethnicity not otherwise specified), and family income, are sourced from student, parent, or school reports, is reported by the student, parent, or the school and gathered from public school records via the North Carolina Department of Public Instruction (for detailed information, please see https://childandfamilypolicy.duke.edu/north-carolina-education-research-data/).

Students in grades 3 through 8 underwent annual assessments in reading and math from 1995 to 2018. Each grade has a dedicated file, containing a single entry for every student enrolled during the testing period. The dataset encompasses individual student test records and provides insights into the testing process, including any modifications made during testing. Additionally, it includes information about students' coursework, birthdates, race, gender, family income backgrounds, learning disabilities, attendance, retests, and exceptional status.

We construct a student-level dataset, incorporating academic test scores (mathematics and reading), and demographic information, such as race and family income. These individual-level datasets were then merged with school-level files, which contained school-level $PM_{2.5}$ exposure and temperature data. This integration allowed for the construction of the dataset used in our primary analysis.

© 2023 Lam PH et al. *JAMA Network Open*

In the initial dataset before data cleaning, we had a total of 11,630,918 student-year level records, representing 2,840,968 individual students. To adhere to the requirements of the two-way fixed-effects model, which necessitates the presence of subjects across different years, we excluded students who appeared only in a single year. Additionally, records lacking information regarding race, family income, or gender were removed. After this cleaning process, we retained 10,346,223 student-year level records. Our analysis primarily focuses on the impact on mathematics and reading scores. Therefore, records with missing values in the outcome variables were also excluded. For mathematics, we retained 10,346,217 student-year level records, encompassing 2,801,021 students. In the case of reading, we retained 10,345,879 student-year level records, representing 2,800,989 students.

Randomization test method

To ascertain the robustness of our core findings and to safeguard against potential model misspecification, we implemented a spatial randomization test. This placebo test entails the random assignment of "false PM2.5" location values to our true timing of the treated sample across 2000 iterations. If the placebo test outcome falls outside the 99% confidence interval of the true estimate, it effectively demonstrates that our estimates are predominantly influenced by the treatment effect, rather than being unduly affected by other time-varying factors.

© 2023 Lam PH et al. *JAMA Network Open*
