# Supplementary material for: Long-Term Exposure to Fine Particulate Matter and Academic Performance Among Children in North Carolina
Source: JAMA Netw Open. 2023 Oct 31;6(10):e2340928. doi: 10.1001/jamanetworkopen.2023.40928 (PMC10618843; doi:10.1001/jamanetworkopen.2023.40928)
Supplement: Supplement 2. — Data Sharing Statement [file jamanetwopen-e2340928-s002.pdf]

Data Sharing Statement

Lam. Long-Term Exposure to Fine Particulate Matter and Academic Performance Among Children in North Carolina. *JAMA Netw Open*. Published October 31, 2023. doi:10.1001/jamanetworkopen.2023.40928

Data
Data available: No

Additional Information
Explanation for why data not available: Due to the data use agreement, we are not allowed to share any student data.
